# Supplementary material for: Function of miR825 and miR825* as Negative Regulators in Bacillus cereus AR156-elicited Systemic Resistance to Botrytis cinerea in Arabidopsis thaliana
Source: Int J Mol Sci. 2019 Oct 11;20(20):5032. doi: 10.3390/ijms20205032 (PMC6829492; doi:10.3390/ijms20205032)
Supplement: Supplementary file 1 [file ijms-20-05032-s001.pdf]

Supplementary materials:

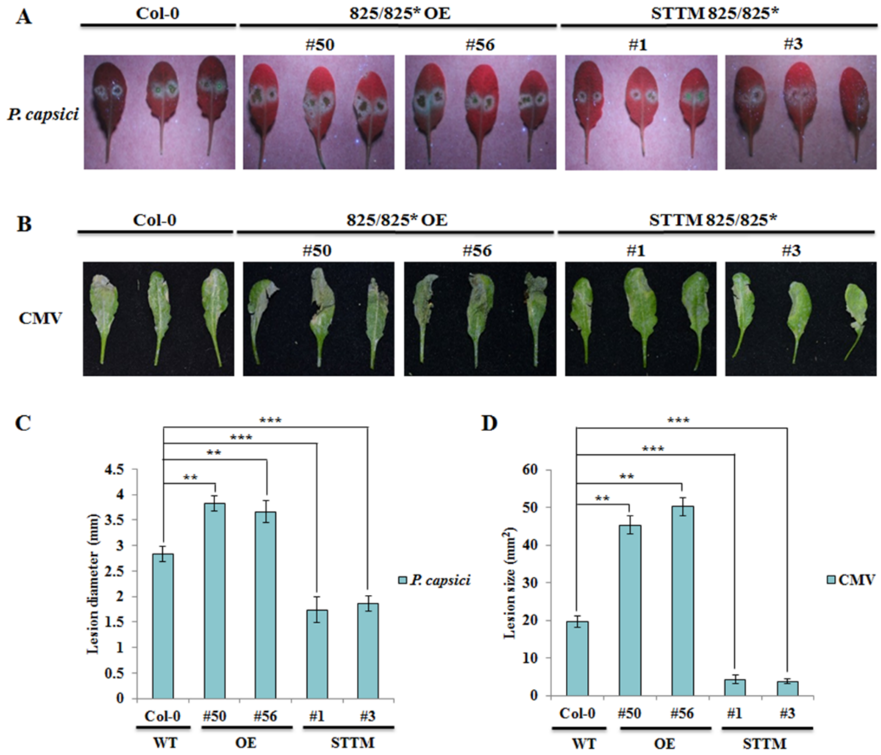

Figure S1. Plant resistance to *P. capsici* and CMV increased in STTM825/825* lines, but decreased in miR825/825* OE lines in comparison with that in Col-0. Three-week-old plants of *Arabidopsis* Col-0, miR825/825* OE lines (#50 and #56), and STTM825/825* lines (#1 and #3) were used for experiments. Approximately 100 *Phytophthora capsici* zoospores were inoculated onto the center of a leaf. Cucumber mosaic virus (CMV) was rub-inoculated onto *Arabidopsis* leaves. (A) Representative photographs of leaves and (D) sizes of leaf necrotic lesions of all tests plant lines inoculated with *P. capsici*, and the photos were taken at 36 hpi. (B) Symptoms and (C) sizes of lesions on leaves of each tested plant line inoculated with CMV at 7 dpi. Student's *t*-test was performed to compare the differences in plants with different genotypes. The data are presented as mean ± SD from three biological replicates. The experiment was repeated three times with similar results obtained. ** $P < 0.01$; *** $P < 0.001$.

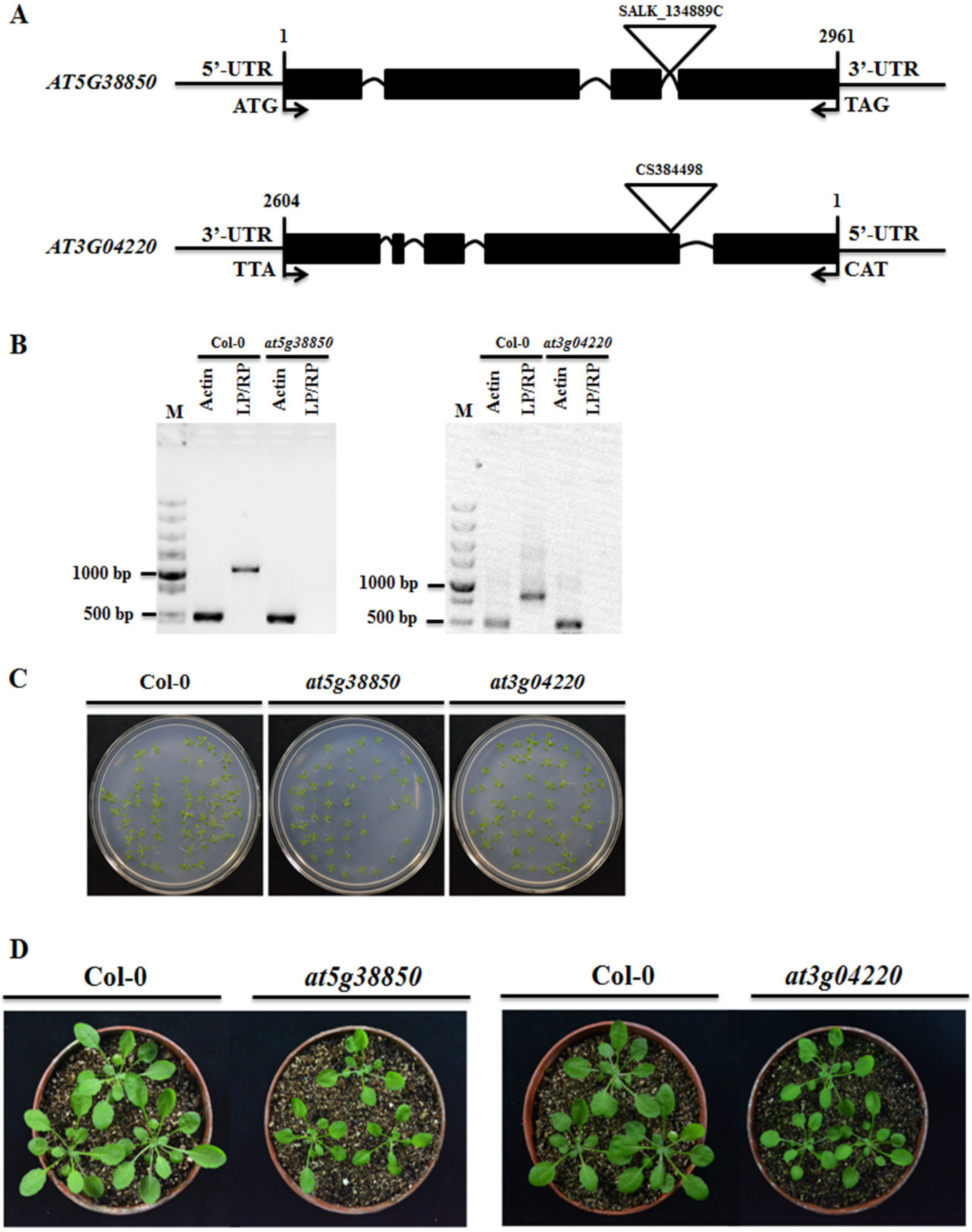

Figure S2. Analysis of miR825 and miR825* target mutants. (A) Structure of the genes *at5g38850* (SALK_134889C) and *at3g04220* (CS384498) with T-DNA insertion sites indicated. Black boxes: exons; curved lines: introns; lines: UTRs; triangles: T-DNA insertion sites. Nucleotide positions are indicated by numbers. (**B**) T-DNA insertions in *at5g38850* (SALK_134889C) and *at3g04220* (CS384498) were confirmed by semi-quantitative RT-PCR, and left arm primer (LP) and right arm primer (RP) were utilized. *AtActin1* was used as the control, with AtActin1-F and AtActin1-R employed as primers. (**C**) Germination of 7-day-old *at5g38850* and *at3g04220* mutant seeds

compared with that of Col-0 plants. (**D**) Phenotypes of four-week-old plants of *at5g38850* and *at3g04220* mutants compared with Col-0 plants.

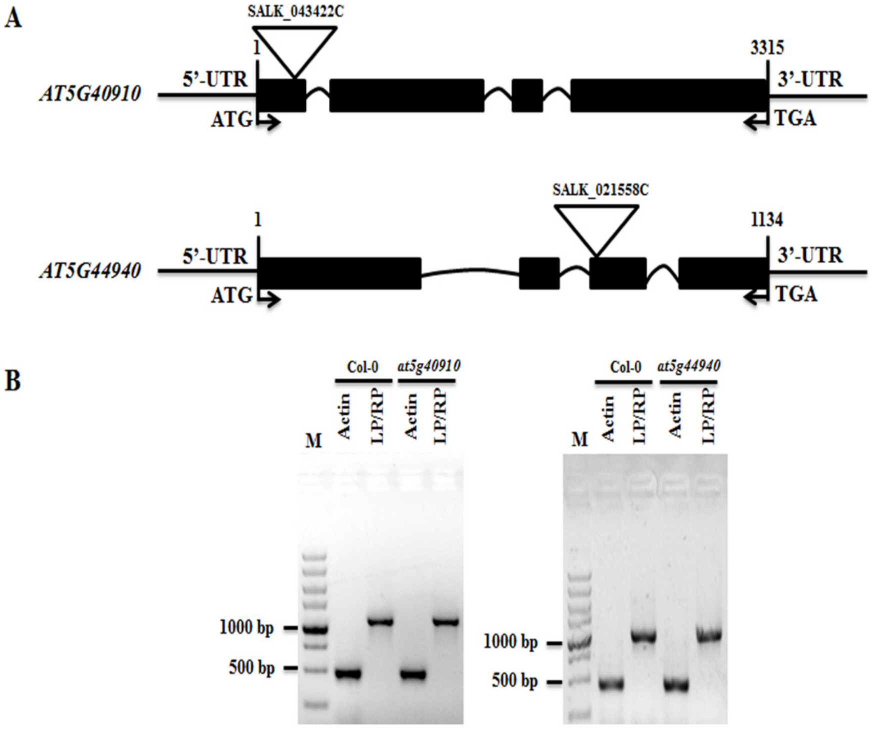

Figure S3. Analysis of *at5g40910* and *at5g44940* mutants. (**A**) Structure of the genes *at5g40910* (SALK_043422C) and *at5g44940* (SALK_021558C) with T-DNA insertion sites indicated. Black boxes: exons; curved lines: introns; lines: UTRs; triangles: T-DNA insertion sites. Nucleotide positions are indicated by numbers. (**B**) T-DNA insertions in *at5g40910* (SALK_043422C) and *at5g44940* (SALK_021558C) were confirmed by semi-quantitative RT-PCR, and left arm primer (LP) and right arm primer (RP) were utilized. *AtActin1* was used as the control, with AtActin1-F and AtActin1-R employed as primers.

Table S1. List of probes and primers used in the current research.

**Probes for small RNA gel blots**	
**Name**	**Sequence (5'–3')**
anti-miR825	GTTCATGCACCTTCTTGAGAA
anti-miR825*	GCTTCTTCGAGCTGGTGCTTGA
anti-U6	TGTATCGTTCCAATTTTATCGGATGT
**Primers for quantitative RT-PCR**	
**Name**	**Sequence (5'–3')**
PR1-F	CTCGGAGCTACGCAGAACAA
PR1-R	TTCTCGCTAACCCACATGTTCA
PR2-F	CGGTACATCAACGTTGGAA
PR2-R	GCGTAGTCTAGATGGATGTT
PR5-F	ATGGCAAATATCTCCAGTATTCACA
PR5-R	ATGTCGGGGCAAGCCGCGTTGAGG
PDF1.2-F	AGTTGTGCGAGAAGCCAAGT
PDF1.2-R	GTTGCATGATCCATGTTTGG
AtActin1-F	GGCGATGAAGCTCAATCCAAACG
AtActin1-R	GGTCACGACCAGCAAGATCAAGACG

BcActin-F	TCCAAGCGTGGTATTCTTACCC
BcActin-R	TGGTGCTACACGAAGTTCGTTG
FRK1-F	GCCAACGGAGACATTAGAG
FRK1-R	CCATAACGACCTGACTCATC
WRKY53-F	CACCAGAGTCAAACCAGCCATTAC
WRKY53-R	CTTTACCATCATCAAGCCCATCGG
miR825* target BETA-TUB4-F	GAGGGAGCCATTGACAACATCTT
miR825* target BETA-TUB4-R	GCGAACAGTTCACAGCTATGTTCA
miR825* target AT5G40910-F	TGTCTTGGCAAATGAAACAGGTACT
miR825* target AT5G40910-R	AACCCCAATAAAGTAACCTTAGACG
miR825* target AT5G38850-F	TTCTCCCTTATTTTGTCCTCGCATT
miR825* target AT5G38850-R	TCTCCTTTCACTGATTTCACCTGTT
miR825* target AT3G04220-F	CAAGGAAGAAATGGGTCAAACAGTG
miR825* target AT3G04220-R	CCATCCCAATCAAATCGTCAAAATC
miR825 target AT5G44940-F	GAGAAGCTCATCTCAGGATGTC
miR825 target AT5G44940-R	CGAGTAACAGATGATCGTATGTCT
**Primers for plasmid construction**	
**Name**	**Sequence (5'–3')**
AT5G40910-F	CGGGGTACCATGGCTTTTTCATCACCGTCTG
AT5G40910-R	CGCGTCGACTCATTGCATCTGAAGAAAGAAGCAAG
AT5G38850-F	CGGGGTACCATGGCTTCTGCTCGAACTTG
AT5G38850-R	CGCGTCGACCTATTTCATATTCATTTCTCCTTTCACTGATTTC
AT3G04220-F	CGGGGTACCATGGATTCTTCTTTTTTACTCGAAACTG
AT3G04220-R	CGCGTCGACTTAGCATTTATAAAACTTCAATCTCTTGAGATAAG
AT5G44940-F	CGGGGTACCATGGAGGAGTTCGATTATATCAGTG
AT5G44940-R	CGCGTCGACTCAATCGATGATAATTTGACATGTGG
**Primers for target mutants**	
**Name**	**Sequence (5'–3')**
LBb1.3	ATTTTGCCGATTTCGGAAC
at5g40910-LP	GAAGCTGACGCCTATGTATGC
at5g40910-RP	CTAAGCCGAAAACCGGTAGAG
at5g38850-LP	CAACATGTGGGTAGACAAGCC
at5g38850-RP	GGCAAGATACGCTCTCAAGTG
at3g04220-LP	CCAAGATCGCAATCATCTTGC
at3g04220-RP	CATAGTAGTCATCGGAACACG
at5g44940-LP	TCGATTAATGGAGTTGATCGG
at5g44940-RP	GACATGTGGGAGAAAGCTTTG
